# I DARE: IULM Dataset of Affective Responses

**DOI:** 10.3389/fnhum.2024.1347327

**Published:** 2024-03-20

**Authors:** Marco Bilucaglia, Margherita Zito, Alessandro Fici, Chiara Casiraghi, Fiamma Rivetti, Mara Bellati, Vincenzo Russo

**Affiliations:** ^1^Behaviour and Brain Lab, Neuromarketing Research Center, Università IULM, Milan, Italy; ^2^Department of Business, Law, Economics and Consumer Behaviour “Carlo A. Ricciardi”, Università IULM, Milan, Italy

**Keywords:** Affect Detection, affective database, Neuromarketing, Consumer Neuroscience, machine learning, EEG, physiological signals

## 1 Introduction

### 1.1 Consumer neuroscience and neuromarketing

Consumer Neuroscience and Neuromarketing apply neuroscience tools and methodologies to investigate consumer behavior (Karmarkar and Plassmann, 2017; Lim, 2018), addressing the limitations of traditional marketing techniques (Hakim and Levy, 2018). Although often used interchangeably, these two terms have different meanings: Consumer Neuroscience, which is more theoretical/academic, is considered as the scientific foundation of Neuromarketing, a more practical and commercially oriented discipline (Nemorin, 2016; Ramsøy, 2019).

Established in the early 2000s (Smith, 2002; Yoon et al., 2006), Neuromarketing and Consumer Neuroscience have become increasingly popular topics among researchers. According to a recent review (Casado-Aranda et al., 2023), as of 2022, 862 articles were published in international journals belonging to psychology (28.45%), business (24.75%), communication (24.08%), and neuroscience (15%), along with management, information technology, and behavioral science (7.72%). The scientific production increased annually by 7.5% and involved 2,354 contributors (3.83 average co-authors per study) with 24.97% of international collaborations.

Consumer Neuroscience and Neuromarketing collect neurophysiological and biometric signals (decoded into “neurometrics”) from subjects during the exposure to marketing stimuli/tasks, in order to assess the underlying cognitive and emotional processes (Cherubino et al., 2019).

The most frequently recorded signals include electroencephalogram—EEG (56.14%), eye tracking—ET (10.53%), skin conductance—SC, cardiac activity (8.77%), and electromyogram - EMG (1.75%) (Rawnaque et al., 2020). Facial expressions are also included in the standard set of Neuromarketing and Consumer Neuroscience measures (Mancini et al., 2023). Typical stimuli include video advertisements (Zito et al., 2021; Russo et al., 2022, 2023), radio commercials (Russo et al., 2020), product packaging (Russo et al., 2021), static creativities (Ciceri et al., 2020), and websites (Bengoechea et al., 2023). Common tasks include the buying processes (Cherubino et al., 2017), in-store experiences (Balconi et al., 2021), and food choices (Stasi et al., 2018).

Decision-making, memory, attention, and approach-withdrawal motivation are the main assessed psychological processes (Cherubino et al., 2019), but emotions, above all, have attracted the greatest scientific interest (Casado-Aranda et al., 2023), probably due to their well-established role in mediating several cognitive functions (Pessoa, 2008; Lerner et al., 2015; Soodan and Pandey, 2016; Tyng et al., 2017).

Neurometrics were originally intended as invariant and highly selective “biomarkers”, as they derived from a large number of experimental neuroimaging studies (Plassmann et al., 2012). However, they have soon been called into question due to their correlational (rather than causal) nature (Plassmann et al., 2015; Ramsøy, 2019), which leads to the reverse inference fallacy (Lee et al., 2017), a common albeit serious problem in neuroscience (Poldrack, 2006). Despite Machine Learning models have been suggested as a reliable solution for the reverse inference issue, they still remain largely ignored (Nathan and Pinal, 2017). Indeed, as of 2019, only 35.1% of Neuromarketing and Consumer Neuroscience studies employed these techniques (Rawnaque et al., 2020).

### 1.2 Affect Detection

Affect Detection (AD) aims to design automatic systems to recognize human emotions from neurophysiological, biometric, and behavioral signals (Calvo and D'Mello, 2010). Initially developed within the Affective Computing, a Computer Science subbranch established in the late 1990s (Picard, 1995, 1997), AD had a far-reaching impact, influencing several research contexts, such as Transportation, Defense, and Secutity, Human Resources, Education, Mental Health and even Market Research (Hernandez et al., 2021).

Since its original formulation, AD has been treated as a Machine Learning problem (Picard, 1995) based on a model *f* trained to predict the affective state *s* from a set of neurophisiological and biometric variables $\text{m}={\left(m_{1},...,m_{m}\right)}^{T}\in {\mathbb{R}}^{m}$, categorized into “modalities” (Picard, 1997). Depending on the chosen emotional theory (Gunes and Pantic, 2010), the emotional state *s* is modeled as a discrete or continuous quantity, which, in turn, determines whether the problem will be treated as a regression or a classification, respectively. A comprehensive discussion on the Machine Learning theory applied to the emotion recognition field goes beyond the scope of this article. Interested readers can refer to introductory tutorials, such as Zhang et al. (2020) and Can et al. (2023).

The classification approach is preferred, even in the case of a continuous emotional model, where the dimensions are early categorized (e.g., low, medium and high). Despite 63% of the studies referred to the continuous emotional models, 92% of them adopted classifiers (D'Mello and Kory, 2015), regardless of the modalities (90% with the voice and 92% with physiological signals) (Imani and Montazer, 2019) and within the wearable context (98%) (Schmidt et al., 2019). Furthermore, a more recent survey that examined more than 380 AD papers published between 2000 and 2020 did not report any regression methods (Wang et al., 2022).

Common modalities include facial expressions, voice, gaze movements, and physiological signals related to either the Peripheral (PNS) or the Central Nervous System (CNS) (Calvo and D'Mello, 2010). Signals from the PNS include EMG, SC, Electrocardiogram (EEG), Photoplestimogram (PPG), and Electrooculogram (EOG). Signals from CNS include EEG, functional Magnetic Resonance Imaging (fMRI), and functional Near-Infrared Spectroscopy (fNIRS) (Shu et al., 2018; Larradet et al., 2020). Voice and facial expressions are the most popular, varying from 82% and 77% (D'Mello and Kory, 2015) to 21% and 54% (Wang et al., 2022), respectively. Physiological signals are adopted in ~16% of the models overall (Wang et al., 2022): at least 10% from the PNS and 5% from the CNS (D'Mello and Kory, 2015). Wearable applications draw a completely different scenario: the modalities include 100% PNS signals, 15% CNS, only 2% facial expressions, and never the voice (Schmidt et al., 2019). Multimodal models (i.e., based on at least two modalities) vary from 85% (D'Mello and Kory, 2015) to 93% (Schmidt et al., 2019).

Affective datasets are collections of modalities annotated in terms of the corresponding affective states and are used to train AD models (D'Mello et al., 2017). The modalities can be either extracted from existing data (e.g., facial expressions from video clips) or newly recorded during experiments (e.g., EEG data from a sample of subjects). Furthermore, affective states can be induced by external stimuli or emerge naturally, as well as posed or spontaneously emitted (D'Mello and Kory, 2015; Zhao et al., 2021).

In the literature, multiple affective datasets that vary largely in terms of modalities, labels, and origin of the affective state are found. We have taken into account a representative sample of 58 datasets, gathered from two reviews (Siddiqui et al., 2022; Ahmed et al., 2023) and three recent research studies (Chen et al., 2022; Saganowski et al., 2022; Pant et al., 2023). Accordingly, the number of modalities varies from 1 to 7 (M = 2.28, SD = 1.24) and include 71.2% the face, 54.2% the voice, 23.7% EEG, 20.3% ECG, 17% PPG, 6.8% EMG, and 6.8% ET. Overall, 81% of the datasets contain newly recorded modalities. The affective states, that are mostly labeled according to a discrete model (70.7%), are mainly induced (69%) and spontaneous (63%).

A recent survey (Ahmad and Khan, 2022) compared the stimuli, modalities, and sample size of the eight most popular datasets for the training of physiological-based AD models: AMIGOS (Miranda-Correa et al., 2018), ASCERTRAIN (Subramanian et al., 2016), BIO-VID-EMO DB (Zhang et al., 2016), DEAP (Koelstra et al., 2011), DREAMER (Katsigiannis and Ramzan, 2017), MAHNOB-HCI (Soleymani et al., 2011), MPED (Song et al., 2019), and SEED (Zheng et al., 2017). The stimuli, varying from 10 to 40 (M = 23.5, SD = 9.943) are all induced and spontaneous. The number of modalities, varying from 1 to 6 (M = 3.38, SD = 1.60), includes EEG (87.5%), ECG (75%), SC (37.5%), respiration (37.5%), PPG (25%), EMG (25%), peripheric temperature—Temp (12.5%), and ET (12.5%). The sample size ranges from 15 to 86 (M = 38, SD = 23.43). The “dataset dimension”, defined as the product between stimuli, modalities, and sample sizes, ranges from 225 to 7,680 (M = 3,257.88, SD = 2,581.12). Finally, the stimuli are all dynamic, including video clips (37.5%), film clips (37.5%), movie clip (12.5%), and music video (12.5%). The features of the eight most popular datasets (plus the proposed one) are presented in Tables 1, 2.

**Table 1:** Qualitative comparison between I DARE and the eight most popular physiological-based datasets in terms of modalities and stimuli type.

**Dataset**	**Modalities**	**Stimuli**
AMIGOS	EEG, ECG, EDA	Video clips
ASCERTRAIN	EEG, ECG, EDA	Movie clips
BIO-VID-EMO DB	ECG, EMG, SC	Film clips
DEAP	EEG, EDA, EMG, PPG, EOG, Resp	Music videos
DREAMER	EEG, ECG	Film clips
MAHNOB-HCI	EEG, ECG, SC, Resp, Temp, ET	Video clips
MPED	ECG, EEG, PPG, SC, Resp	Video clips
SEED	EEG	Film clips
I DARE	EEG, EMG, SC, PPG, ET	Images

**Table 2:** Quantitative comparison between I DARE and the eight most popular physiological-based datasets in terms of number of modalities, stimuli, and subjects, as well as dimension and minimum detectable effect size (Cohen's *f* ).

**Dataset**	**# Modalities**	**# Stimuli**	**# Subjects**	**Dimension**	**Cohen's *f***
AMIGOS	3	16	40	1,920	0.149
ASCERTRAIN	3	36	58	6,264	0.095
BIO-VID-EMO DB	3	15	86	3,870	0.104
DEAP	6	40	32	7,680	0.125
DREAMER	2	18	23	828	0.191
MAHNOB-HCI	5	20	27	2,700	0.170
MPED	4	28	23	2,576	0.166
SEED	1	15	15	225	0.253
I DARE	5	32	63	10,080	0.095

For each dataset, we conducted statistical analyses using JASP (Love et al., 2019), to explore relationships between the number of modalities, stimuli, and sample size. Additionally, we performed sensitivity analyses using G*Power (Faul et al., 2007) to estimate, from the sample size and the number of stimuli, the minimum detectable effect size. No significant correlations between the number of modalities and subjects (Spearman's correlation: ρ = 0.270, *p* = 0.518), modalities and stimuli (Spearman's correlation: ρ = 0.700, *p* = 0.053), and subjects and stimuli (Spearman's correlation: ρ = 0.084, *p* = 0.843) emerged, suggesting that the dataset-specific characteristics are not bounded by technological or experimental constrains. The sensitivity analysis, which was based on the repeated measures ANOVA model (stimulus ID as within factor, α = 0.05, 1−β = 0.95, *r* = 0.5, ϵ = 1), showed an average minimum detectable effect size of *f* = 0.157 ± 0.051 ranging from 0.095 to 0.253, interpretable as “small” to “medium” (Cohen, 2013).

### 1.3 I DARE dataset

We contend that the diffusion of AD models within Neuromarketing and Consumer Neuroscience could be increased by addressing the limitations of existing datasets. We believe that the strictest one is the use of videos as a unique elicitation method, which may lead to AD models that are unsuitable for the evaluation of static or short-term stimuli (e.g., static creativities, product packaging, or landing pages), as well as for a dynamic classification (Bilucaglia et al., 2021) of multi-frame stimuli (e.g., video commercials). A less severe (but still present) limitation is the varying number of stimuli that affects the minimum detectable effect size and, in turn, the reliability and generalizability of the results (Funder and Ozer, 2019). Finally, despite their popularity in Consumer Neuroscience and Neuromarketing, modalities such as ET, SC, PPG and EMG are not as widely represented.

We present I DARE, the IULM Dataset of Affective REsponses, a multimodal (five modalities: EEG, SC, PPG, EMG, and ET) physiological affective dataset gathered from 63 participants, based on 32 images as elicting stimuli, corresponding to a dimension of 5 × 32 × 63 = 10, 080. A sensitivity analysis, performed using G*Power under the same previously described conditions, showed a minimum detectable effect size of *f* = 0.095, considered as “small”. As shown in Table 2, I DARE exceeds existing dataset in three key aspects: it is based on static pictures, it has the highest dimension, and it allows the lowest minimum detectable effect size.

## 2 Materials and methods

### 2.1 Stimuli selection

We extracted the eliciting stimuli from both the IAPS (Lang et al., 2008) and OASIS (Kurdi et al., 2017), two standard datasets of realistic pictures that have been annotated in the valence and arousal dimensions by ~100 independent raters. The annotations are provided as mean (*m*) and standard deviation (*s*) across the raters, for both the valence (*m*_*v*_, *s*_*v*_) and the arousal (*m*_*a*_, *s*_*a*_) dimensions.

For each dataset, we performed the stimuli selection through a seven-step semi-automatic procedure. Steps 1–5 (automatic) were based on the geometrical properties of the stimuli in the two-dimensional Valence-Arousal space, which were considered either as points $\text{x}={\left(m_{v},m_{a}\right)}^{T}$ or gaussian vectors x~N(μ,Σ), with $\mu ={\left(m_{v},m_{a}\right)}^{T}$ and $\Sigma =\left(\begin{array}{cc} s_{v} & 0\\ 0 & s_{a} \end{array}\right)$. Steps 6, 7 were based on the ratings provided by nine independent judges.

The selection process yielded 32 stimuli (16 per dataset), belonging to each of the 4 quadrants of the Valence-Arousal space (HVLA - High Valence High Arousal, HVLA - High Valence Low Arousal, LVHA—Low Valence High Arousal, and LVLA - Low Valence Low Arousal). The whole procedure is shown in the https://figshare.com/projects/I_DARE/186558 (Stimulus_Selection.pdf file).

### 2.2 Sample population and experimental protocol

Sixty-three people (24 men) with age ranging from 20 to 30 years (M = 22.89, SD = 2.38) were enrolled in the study. Statistical analyses performed using JASP showed that the sample was not significantly gender unbalanced in terms of proportions (Binomial test: Male = 38.1%, *p* = 0.077) and mean age (Mann–Whitney U test: *W* = 3995, *p* = 0.325). The sample characteristics are shown in https://figshare.com/projects/I_DARE/186558 (Sample.csv file).

The protocol was approved by the Ethics Committee of the Università IULM, and an informed consensus was obtained from each participant.

Each subject sat on a chair placed in front of a 23.8^′′^ (527 × 296.5*mm*^2^) FlexScan EV2451 (Eizo, KK) monitor located in a 7 × 3*m*^2^ room. At the beginning of the experiment, the subject performed a 60 s-long eye-closed baseline and a 120 s-long neutral baseline (white fixation dot on a black background). This was preceded by the baseline instructions and followed by the experiment description. Then, 32 emotional blocks were presented in a random order. Each block consisted of a black screen (5 s), followed by the emotional stimulus (5 s) and the valence/arousal evaluation, performed by means of a nine-point SAM (Bradley and Lang, 1994). The description and duration of the stimuli are shown in https://figshare.com/projects/I_DARE/186558 (Stimuli_Specification.csv file).

The quality of the signals was visually checked before starting the recording, and the contact impedance of the EEG electrodes was reduced below 10*kΩ* (Sinha et al., 2016) by means of a scrub cream (Neurprep by Spes Medica, S.p.A.) and a conductive gel (Neurgel by Spes Medica, S.p.A.).

### 2.3 Instrumentation

We recorded the EEG signal using the NVX-52 device (Medical Computer Systems, Ltd.) at a sample frequency of *f*_*s*_ = 1*kHz*. Forty passive wet Ag / AgCl electrodes were placed at standard locations (Russo et al., 2023) of the 10-10 system (Nuwer, 2018) and referenced to the left mastoid (M1) by means of 1 Ag/AgCl passive adhesive patch. We recorded the SC and PPG signals using the GSRSens and FpSens (Medical Computer Systems, Ltd.) sensors, respectively, both connected to the auxiliary inputs of the NVX-52. We placed two passive dry Ag/AgCl electrodes of the GSRSens on the index and middle finger from the non-dominant hand and the FpSens on the ring finger from the same hand. We recorded the EMG signal using two passive Ag/AgCl adhesive patches placed over the left *zygomaticus major* and *corrugators supercilii* muscles. The electrodes, connected to the bipolar inputs of the NVX-52, were referenced to M1. The recordings were controlled by NeoRec software (Medical Computer Systems, Ltd.). We recorded the ET signal using the Tobii Spectrum (Tobii, LLC) device at a sample frequency of *f*_*s*_ = 150*Hz*. The collected measures included the horizontal and vertical coordinates of the right and left eyes (*x*_*R*_, *y*_*R*_, *x*_*R*_, and *y*_*L*_, respectively), expressed in *mm* with respect to the upper-left corner of the screen.

We used the iMotions software (iMotions, A/V) to deliver the stimuli and to collect the eye tracking data and the SAM responses. At the beginning of the experiment, iMotions generated a TTL pulse that was fed into the digital inputs of the NVX-52 using the ESB - EEG Synchronization Box (Bilucaglia et al., 2020). This served to perform an off-line synchronization between the recorded data and the stimuli timestamps.

### 2.4 Data processing

We aligned the recorded data with the TTL pulse and appended the stimuli, according to the iMoions-generated timestamps. Then, we cleaned the data from noise and artifacts, as described below. The operations were performed within the Matlab (The Mathworks, Inc.) environment, and the processed data were saved as .mat files. A comprehensive discussion on the biomedical data processing goes beyond the scope of this article. Interested readers can refer to introductory textbooks, such as Blinowska and Żygierewicz (2021) and Rangayyan and Krishnan (2024).

#### 2.4.1 EEG

We processed the EEG data using the EEGLab toolbox (Delorme and Makeig, 2004). We applied a re-reference to the linked earlobes, a resample to *f*_*S*_ = 512*Hz*, a band-pass filter (0.1 − 40*Hz*, 4*th* order zero-phased Butterworth filter), and an adaptive filter (cleanline plug-in), to remove the sinusoidal noise (50*Hz*, 100*Hz*). We applied the artifact Subspace Reconstruction (cutoff parameter *k* = 20) to correct large non-stationary artifacts (Chang et al., 2018). Then, we performed the Independent Component Analysis (ICA) decomposition trough the RunICA algorithm (Akhtar et al., 2012) on a copy of the original data that have been band-pass filtered (1 − 30*Hz*, 4*th* order zero-phased Butterworth filter) and down-sampled (*f*_*s*_ = 100*Hz*) (Luck, 2022). The obtained ICA weights were then saved and multiplied to the original data, to get the ICs. We identified artifactual ICs through the neural-net classifier ICLabel (Pion-Tonachini et al., 2019) as those with probability of “non-brain” P{!brain}>0.9, and we set them to zero and back-projected them to the original space. Finally, we re-referenced the artifact-free data to the approximatively-zero potential REST (Dong et al., 2017).

#### 2.4.2 SC

We applied a resample at *f*_*S*_ = 32*Hz* and a band-pass filter (0.001 − 0.35*Hz*, 4*th* order zero-phased Butterworth filter). We identified artifactual points as those exceeding two fixed thresholds (amplitude: 0.05–60 μS, rate of change: ±8 μS/s) and deleted all the neighbour within a centered 5*s*−long window (Kleckner et al., 2018). Finally, we reconstructed the deleted points by means of a linear interpolation applied on the whole data.

#### 2.4.3 PPG

We applied a resample at *f*_*S*_ = 32*Hz*, a band-pass filter (0.001 − 0.35*Hz*, 2*nd* order zero-phased Butterworth filter), and a filter based on the Hilbert's transform, to attenuate slow drifts and motion artifacts (Dall'Olio et al., 2020).

#### 2.4.4 EMG

We applied a notch filter (50*Hz*, 100*Hz, q* = 40, zero-phased IIR filter), to remove the power-line noise and a band-pass filter (10 − 400*Hz*, 4*th* order zero-phased Butterworth filter), to attenuate low-frequency motion artifacts (Merletti and Cerone, 2020).

#### 2.4.5 ET

We corrected temporal jitters by re-aligning the timestamps with the equipment-designed sample frequency (*fs* = 150*Hz*) and linearly interpolating the missing values (Liu et al., 2018). Then, we filtered the coordinates *x*_*R*_, *y*_*R*_, *x*_*L*_, and *y*_*L*_ using the MFLWMD algorithm (window length 140*ms*, threshold *g* = 280*ms*), to reduce outliers and missing data (Gucciardi et al., 2022). Finally, we computed the horizontal and vertical gaze positions as, respectively, *g*_*X*_ = (*x*_*R*_+*x*_*L*_)/2 and *g*_*Y*_ = (*y*_*R*_+*y*_*L*_)/2 (Kar, 2020).

## 3 Dataset description

I DARE includes the output of the stimuli selection process, the SAM evaluations, and the processed modalities.

### 3.1 Stimuli selection

The selected stimuli obtained a percentage agreement ranging from 56.56% to 100% (M = 79.20%, SD = 10.50%). Statistical analyses performed using JASP did not show any significant main effect for the factors “Database” [*F*_(1, 24)_ = 0.143, *p* = 0.709] and “Quadrant” [*F*_(3, 24)_ = 2.381, *p* = 0.095], as well as for the “Database × Quadrant” [*F*_(3, 24)_ = 0.048, *p* = 0.986] interaction. Additionally, the agreement did not significantly correlate with the mean (*m*) and standard deviations (*s*) of the overall ratings in both the valence (ρ_*m*_ = 0.194, *p* = 0.288;ρ_*s*_ = −0.175, *p* = 0.339) and the arousal (ρ_*m*_ = −0.142, *p* = 0.439; ρ_*s*_ = 0.109, *p* = 0.553) dimensions. The results of the stimuli selection process are shown in https://figshare.com/projects/I_DARE/186558 (Agreement_Raters.csv file).

### 3.2 SAM evaluations

We coded the subjects' evaluations (SAM numerical scores) into labels corresponding to the four quadrants (HVLA, LVLA, HVHA, and LVHA), considering the mid-point value 5 as threshold for the low/high discrimination. We measured the overall agreement within the subjects (*WS*) and between the subjects and the gold standard (*GS*) given by the selection process by means of the Fleiss' kappa (Fleiss, 1971). The analyses, performed using JASP, showed values of κ_*WS*_ = 0.406 and κ_*GS*_ = 0.409, both intepreted as a “fair agreement” (Landis and Koch, 1977). The SAM evaluations are shown in https://figshare.com/projects/I_DARE/186558 (Valence_SAM.csv, Arousal_SAM.csv and Quadrants_SAM.csv files).

### 3.3 Modalities

We grouped the processed data based on their sample frequency and stored them into separated folders (EEG, EMG, SC&PPG, and ET) containing 63 .mat files each (1 per subject). The files consist of Matlab's “structure arrays” with the following fields:

- subject: subject's unique identifier
- Fs: sample frequency
- channels: channels' name
- channels_type: modality (EEG, SC, PPG, and ET)
- channels_unit: channels units of measurement (μ*VμS, a*.*u*., *mm*)
- data: data matrix (rows: channels, columns: sample points)
- time: vector of sample points
- event_id: stimuli labels
- event_begin: stimuli onsets as positions within the time vector
- even_end: stimuli offsets as positions within the time vector

## 4 Conclusion

Emotions are the most commonly explored aspect within Consumer Neuroscience and Neuromarketing. Their assessment has been mainly performed by mean of neurometrics, an approach often questioned due to the potential reverse inference problem. By contrast, Machine Learning methods (i.e., AD models), that have been proposed as a reliable solution to the issue, are still under-represented.

Existing physiological datasets rely on dynamic eliciting stimuli, making them unsuitable to train AD models for the evaluation of images or the dynamic frame-by-frame classification of videos. Furthermore, the available data sets are characterized by a wide-varying number of subjects, stimuli, and modalities, negatively affecting their reliability and generalizability.

To fill these gaps, we created I DARE (the IULM Dataset of Affective REsponses), a multimodal physiological dataset that outperforms existing ones in terms of dimension (5 modalities × 32 stimuli × 63 subjects) and minimum detectable effect size (*f* = 0.095, small). We selected the stimuli (static pictures) from the two standard databases (IAPS and OASIS) through a semi-automatic procedure, to reduce the selection biases. We obtained “fair” agreements within subjects and between subjects and the original scores (κ_*WS*_ = 0.406 and κ_*GS*_ = 0.409, respectively).

We must acknowledge some limitations of I DARE. First, while the sample was not significantly gender unbalanced, it mainly consisted of young subjects (i.e., ≤ 30 years old). This could potentially impact the sensitivity of trained AD models toward age differences. Including new data from older subjects would not only help to address the imbalance in the dataset but also increase its size and, consequently, its representativity. Second, the experimental paradigm, which was chosen to minimize confounding effects and biases, may also have affected the ecological validity of the collected data. As a result, the applicability of trained AD models in real-life situations may be limited. To examine this hypothesis, future experiments outside the laboratory, using natural stimuli and wearable devices, should be carried out.

Nevertheless, we believe that I DARE could be helpful in several applications. It could be used for the emotional assessment of static creativities (e.g., product packaging, landing pages) and the dynamic classifications of video commercials in Consumer Neuroscience and Neuromarketing researches, as well as to train AD models for Affective Computing and Neuroergonomics applications. Additionally, it could serve as a benchmark for the assessment of the emotional selectivity and invariance of existing neurometrics, as well as to study the physiology of emotions in the Psychology and Cognitive Neuroscience fields.

To foster its adoption by a larger audience of scientists, I DARE has been made publicly available at https://figshare.com/projects/I_DARE/186558.

## Data availability statement

The datasets presented in this study can be found in online repositories. The names of the repository/repositories and accession number(s) can be found below: https://figshare.com/projects/I_DARE/186558.

## Ethics statement

The studies involving humans were approved by Ethics Committee of Università IULM. The studies were conducted in accordance with the local legislation and institutional requirements. The participants provided their written informed consent to participate in this study. Written informed consent was obtained from the individual(s) for the publication of any potentially identifiable images or data included in this article.

## Author contributions

MBi: Conceptualization, Data curation, Formal analysis, Methodology, Writing—original draft, Writing—review & editing. MZ: Supervision, Writing—review & editing. AF: Methodology, Writing—original draft. CC: Writing—original draft. FR: Investigation, Methodology, Writing—review & editing. MBe: Investigation, Methodology, Writing—review & editing. VR: Supervision, Writing—review & editing.
